# Structural Insight into Paramyxovirus and Pneumovirus Entry Inhibition

**DOI:** 10.3390/v12030342

**Published:** 2020-03-20

**Authors:** Megha Aggarwal, Richard K Plemper

**Affiliations:** Institute for Biomedical Sciences, Georgia State University, Atlanta, GA 30303, USA; maggarwal@gsu.edu

**Keywords:** Respiratory syncytial virus, parainfluenzavirus, measles virus, nipah virus, pneumovirus, paramyxovirus, virus entry, antiviral therapeutic, entry inhibitor

## Abstract

Paramyxoviruses and pneumoviruses infect cells through fusion (F) protein-mediated merger of the viral envelope with target membranes. Members of these families include a range of major human and animal pathogens, such as respiratory syncytial virus (RSV), measles virus (MeV), human parainfluenza viruses (HPIVs), and highly pathogenic Nipah virus (NiV). High-resolution F protein structures in both the metastable pre- and the postfusion conformation have been solved for several members of the families and a number of F-targeting entry inhibitors have progressed to advanced development or clinical testing. However, small-molecule RSV entry inhibitors have overall disappointed in clinical trials and viral resistance developed rapidly in experimental settings and patients, raising the question of whether the available structural information may provide a path to counteract viral escape through proactive inhibitor engineering. This article will summarize current mechanistic insight into F-mediated membrane fusion and examine the contribution of structural information to the development of small-molecule F inhibitors. Implications are outlined for future drug target selection and rational drug engineering strategies.

## 1. Introduction

The paramyxovirus and pneumovirus families belong to the order Mononegavirales (1), which includes additional major human pathogen families such as the filoviruses and rhabdoviruses. Characteristic for the order are lipid-enveloped virions that contain a negative sense non-segmented (NNS) RNA genome. Genomic and antigenomic RNAs are viral nucleoprotein-encapsidated, resulting in the formation of ribonucleoprotein (RNP) complexes [1]. Spreading through the respiratory route, most paramyxo- and pneumoviruses are highly contagious, causing collectively major morbidity and mortality worldwide in particular in pediatric patients, older adults, and among the immune compromised.

For instance, the paramyxoviruses HPIV1 and HPIV3 represent a major health threat to transplant patients [2,3]. HPIV3 alone infects currently up to 18% of hematopoietic stem cell transplant recipients [4]. Approximately 20–40% of these patients develop severe disease with viral spread to the small airways, which coincides with an increase in case-fatality rates from 10% when infection remains restricted to the upper respiratory tract to 27% in patients with viral pneumonia [5,6,7,8]. No approved vaccine prophylaxis or antiviral therapeutic is currently available to protect against any HPIV infection or improve disease management. 

Another example for severe and unmitigated paramyxovirus disease is provided by NiV, a zoonotic member of the family that belongs to the henipavirus genus. The henipaviruses are highly pathogenic in humans and many animals, causing systemic infections with severe neurological complications. In the past two decades, repeated NiV outbreaks in the Asian region originated from transfer of the virus from its natural bat host to domestic animals, resulting in case/fatality rates reaching from 40% to over 90% [9]. Continued spillover into the human population must be expected, and human-to-human transmission through respiratory secretions, urine, and saliva has been documented [10]. 

Of the pneumoviruses, RSV infects almost all children before two years of age and is responsible for over 100,000 hospitalizations yearly in the United States alone. The monoclonal antibody palivizumab has been approved for immunoprophylaxis against RSV infection [11], however, use is restricted to high-risk patients due to the high-cost and need for prophylactic administration. 

Given the health, medical and economic burden associated with paramyxo- and pneumovirus infections, a major and currently unmet clinical need exists to expedite the development of novel safe and effective therapeutics for improved disease management and outbreak control. Current direct-acting therapeutics predominantly focus on preventing viral entry through neutralizing antibodies (nAbs) and on small-molecules targeting the envelope glycoproteins or inhibiting the viral RNA-dependent RNA polymerase (RdRP) complex. Reflecting major efforts to identify a cost-effective alternative to high-price passive immunization with anti-RSV nAbs, a number of compounds have entered advanced preclinical development and clinical testing in recent years (Table 1). Breakthroughs in the structural and functional characterization of the viral entry machinery and polymerase complexes in the past decade [12,13,14,15,16,17,18,19,20,21] have furthermore created a novel opportunity for structure-informed mechanistic characterization and ligand optimization. 

With a focus on pharmaceutical targeting of the viral entry machinery, we will discuss in the following the main stages of pneumo- and paramyxovirus F protein-mediated membrane fusion, examining the conformational rearrangements required for membrane merger. This structural framework of the viral entry process will be overlaid with known neutralizing epitopes and target sites for small-molecule entry inhibitors to better appreciate the underlying mechanism of inhibition, the structural basis for viral resistance, and the potential for counteracting viral escape through proactive ligand engineering. 

## 2. The Pneumo- and Paramyxovirus Entry Machinery

The entry process involves two major steps, attachment and fusion, which in the case of the paramyxoviruses is performed by two distinct protein complexes; the H, HN, or G proteins, depending on the paramyxovirus examined, and the F proteins, respectively. In contrast, pneumovirus F protein function is not strictly dependent on stimulation by an attachment protein in experimental settings. 

### 2.1. Attachment Proteins

**Figure 1:**
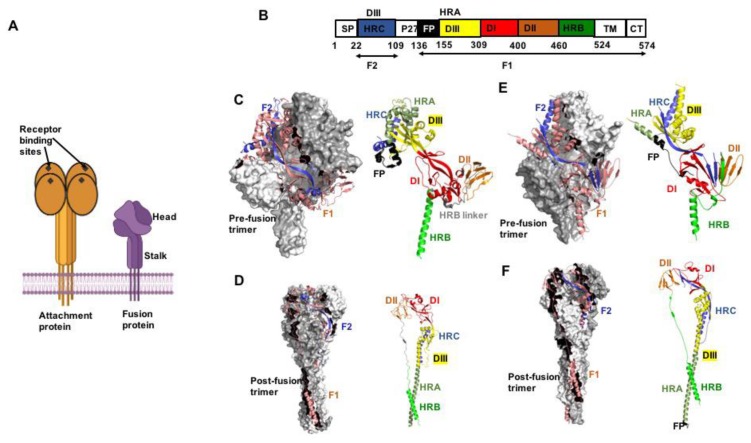
The pneumo- and paramyxovirus entry machinery. (**A**) Schematic of paramyxovirus attachment and F proteins showing the overall head and stalk organization of the attachment protein tetramer and F protein trimer, respectively. (**B**) Color-coded schematic representation of the F protein domain organization (shown by example of RSV F). Heptad repeat (HR) domains A (HRA) and B (HRB) form the post- and prefusion F helical stalks, respectively. HR domain C (HRC) is located in the membrane-distal section of the prefusion F head. SP, signal peptide; FP, fusion peptide; TM, transmembrane domain; CT, cytoplasmic tail. The precursor F_0_ precursor protein is cleaved into F_1_ and F_2_ subunits. (**C**) Crystal structure of the prefusion PIV5 F trimer (PDB 4GIP). Surface view with one monomer shown as cartoon (left panel). F_2_ in blue and F_1_ in salmon. Domain view of a single monomer (right panel), colored as in (B). (**D**) Postfusion hPIV3 F trimer (PDB 1ZTM), the 6HB is oriented towards the base of the structure. (**E**,**F**) Pre- and postfusion forms of RSV F (PDB 4MMQ and 3RRR, respectively). Subtle differences in overall geometry to paramyxovirus F are present in the prefusion head specifically. All figures were prepared with PyMol (DeLano Scientific; http://pymol.sourceforge.net/).

The physiological oligomer of all paramyxovirus attachment proteins is a homo-tetramer, consisting of a dimer-of-dimers. Inserted into the viral envelope in type 2 transmembrane protein orientation, the native attachment protein tetramer consists of membrane-distal globular head domains and a membrane-proximal α-helical coiled-coil stalk (Figure 1A) [1,22]. The head domains of paramyxovirus attachment protein types assume the overall six-bladed propeller fold of sialidases. However, only HN proteins, which are found, for instance, on members of the respirovirus and rubulavirus genera, have actual neuraminidase activity, and only paramyxoviruses with HN-type attachment proteins use sialic acid moieties displayed on the target cell plasma membrane as receptor. In contrast, morbilliviruses such as MeV and henipaviruses enter cells through proteinaceous receptors, CD150 and Nectin-4 in the case of MeV [23,24,25], and ephrin-type receptors in the case of NiV [26,27], which eliminates the need for neuraminidase catalytic activity. The sialidase-like fold of the head domains in nevertheless conserved also across the morbilliviruses and henipaviruses, indicating that sialic acid-mediated attachment to target cells represents the entry strategy of a common ancestral paramyxovirus.

The pneumovirus G protein lacks both hemagglutination and neuraminidase activity. Structural information is very limited compared to that available for the paramyxovirus attachment proteins, but pneumovirus G is conformationally distinct and does not share the sialidase-like fold that is characteristic for paramyxoviruses. Whereas the paramyxovirus attachment proteins are furthermore essential for virion infectivity, recombinant RSV lacking G could be recovered and propagated in cell culture [28,29,30], indicating that RSV F must have both cell attachment and membrane fusion activity. However, RSV G acts as an important virulence factor and ΔG RSV recombinants are apathogenic in vivo [31,32]. The exact mechanistic contribution of RSV G to membrane fusion is unclear, but a functional interaction with RSV F was established experimentally examining homo- and heterotypic F and G proteins [33].

### 2.2. F Proteins

Compared to the attachment proteins, the overall fold of the F protein is very conserved across the two families. F proteins are integral membrane proteins in type 1 topology. They are synthesized as bio-inactive precursor proteins F_0_ that require proteolytic maturation. The physiological oligomer is a homo-trimer, and only the trimer can assume a native conformation and gain intracellular transport competence. Accordingly, newly synthesized F trimerize in the endoplasmic reticulum (ER), and in the case of the Morbilliviruses the ER is also the site of subsequent hetero-oligomerization of attachment and fusion protein complexes (24). F trimers assume initially a metastable prefusion fold that resembles a lollipop-like shape (Figure 1A–F), consisting of a membrane-proximal coiled-coil prefusion stalk and a single globular head domain. Both stalk and head domains are cooperatively formed by all three monomers [1]. The stalk connects to the transmembrane domains and short C-terminal cytosolic tails. Anchoring of the transmembrane domains in the lipid envelope adds stability to the prefusion conformation. Consequently, crystal structures of prefusion PIV5 and NiV F ectodomains could only be obtained when the transmembrane domains were replaced with soluble trimerization domains [21,34]. Crystallization of prefusion PIV3, MeV, and RSV F required additional disulfide bonds and/or cavity filling mutations to stabilize the soluble ectodomains [35,36,37]. 

For most paramyxo- and pneumovirus F proteins, proteolytic maturation is mediated by host cell furin-type proteases resident in the Golgi apparatus. Cleavage generates for each monomer a larger, membrane-embedded F_1_ and shorter F_2_ fragment, which remain covalently connected to F_1_ through an intra-monomeric disulfide bond [1,22]. The newly liberated N-terminus of F_1_ is positioned at the beginning of the fusion peptide, a stretch of largely hydrophobic amino acids that functions as a membrane attack group and is inserted into the target membrane during the fusion process. Directly adjacent to the fusion peptide and to the transmembrane domain are highly conserved amphipathic α-helical regions with a 3-4 heptad repeat (HR) pattern, the HR-A and HR-B domains, respectively [21]. 

In the thermodynamically stable post-fusion conformation, F trimers assume an overall cone-shaped form (Figure 1D,F) that is characterized by a large central triple helix coiled-coil formed by the HR-A domains. In prefusion F, the HR-A helices were broken up into 11 distinct structural elements that formed the membrane-distal section of the prefusion F head [21]. The shorter HR-B domains pack against the grooves of the triple helix, resulting in a thermodynamically highly stable 6-helix bundle (6HB) or fusion core arrangement [18,20,38]. In the 6HB, the HR-adjacent transmembrane domains and fusion peptides, and consequently viral envelope and target membrane, are in direct proximity, residing in the same lipid bilayer. 

### 2.3. Fusion Activation

Triggering of pneumo- and paramyxovirus F proteins for membrane fusion is receptor binding-induced and pH-independent. In the case of the paramyxoviruses, the attachment proteins head domains can assume a pre-receptor bound heads-down or post-receptor heads-up orientation relative to the attachment protein stalk domain. Truncated attachment proteins lacking the head domains were engineered for paramyxoviruses of the rubulavirus, morbillivirus, respirovirus, and henipavirus genus [39,40,41,42]. These attachment protein stems trigger refolding of the homotypic F proteins indiscriminate of receptor binding, indicating that the attachment protein stalks act as the effector domains whereas the attachment protein heads function as activity regulators. 

As a common theme of fusion triggering, receptor binding results in a conformational rearrangement of the attachment protein head domains, which move from heads-down to heads-up position. In the case of the rubula- and respirovirus HN proteins, this change exposes an F interaction site on the HN stalk, resulting in hetero-oligomerization of HN tetramers and F trimers that triggers F refolding [43,44]. By contrast, morbillivirus envelope proteins first hetero-oligomerize in the host cell ER [45], indicating that the heads-down arrangement of the attachment proteins does not sterically mask the F interaction sites on the stalk domains. Rather, receptor binding by morbillivirus H proteins and the associated subsequent head domain rearrangement is thought to initiate conformational changes in the H stalk [46], triggering refolding of the pre-associated F trimers according to a safety-catch model [47]. 

Once the F refolding cascade has been activated either through triggering by the attachment protein or, in the case of the pneumoviruses, direct F interaction with receptor [48,49,50], assembly of the HR-A triple helix propels the fusion peptide towards the target membrane, resulting in linkage of fusion donor and acceptor lipid envelopes through F proteins in extended pre-hairpin conformation. Subsequent concerted hairpin formation by several F trimers and zippering of the 6HB domains locally introduces extreme negative curvature (away from the cytoplasm or virion lumen, respectively) into the lipid bilayers, resulting in local disarray of the lipid layers at the approaching fusion tips that facilitates membrane merger [22,43]. Thus, the F proteins mediate fusion pore opening by acting as membrane bending machines, that are thought to be driven predominantly by the free energy released through 6HB assembly. 

## 3. Entry Inhibition

Counting drugs in clinical use, drug candidates in clinical trials or under formal development, and early-stage experimental compounds, a large number of direct-acting entry inhibitors blocking pneumo- or paramyxovirus infection have been identified. Chemically, these include biopharmaceuticals such as neutralizing antibodies [14,37,51,52,53,54] synthetic peptides [55,56], and chemically synthesized small molecules [57,58,59,60,61]. However, the anti-RSV nAb palivizumab is the only F protein-targeting drug approved for human use, and a number of small-molecule candidates that have advanced to clinical testing are likewise directed against RSV. The RSV indication therefore provides the richest dataset to examine the strengths and challenges of therapeutic targeting of F proteins. 

### 3.1. Synthetic F Protein Blockers

Two predominant mechanisms of productive interference with F protein function by synthetic antivirals have emerged: late-stage inhibition of fusion core assembly with peptidic inhibitors and stabilization of a prefusion F conformation with small-molecule compounds. Most synthetic anti-F peptides are derived from residues of the homotypic or a closely related HR-B domain and block 6HB closure through competition with endogenous HR-B for access to the HR-A triple helix grooves [62]. Consequently, these peptides act late in the F refolding cascade, trapping the trimer in a pre-hairpin conformation, analogous to the mechanism of action proposed for the only clinically approved direct-acting antiviral peptide, the HIV entry inhibitor Fuzeon [63,64]. Although efficacious, Fuzeon also embodies the liabilities of HR-derived therapeutic peptides [65]: the drug product is difficult to manufacture resulting in high treatment cost, oral bioavailability is lacking, and the virus-derived peptides are immunogenic, causing severe injection site reactions [66]. Reflecting these limitations of synthetic peptides, advanced preclinical development and clinical trials have mostly focused on small-molecule antivirals. 

**Figure 2:**
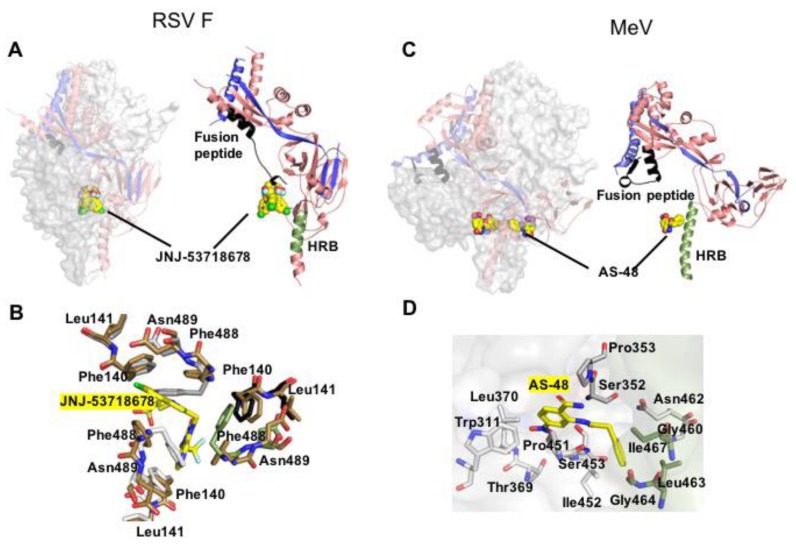
Prefusion pneumo- and paramyxovirus F structures complexed with small molecule inhibitors. (**A**) JNJ-53718678 (shown as yellow sphere) bound to RSV F (PDB 5KWW). The compound interacts with the base of the central F cavity and the fusion peptide, stabilizing the prefusion conformation. (**B**) Close-up of the hydrophobic binding pocket of JNJ-53718678 (yellow sticks) that involves all the three monomers of the F trimer. (**C**) MeV F in complex with small molecule entry inhibitor AS-48 (PDB 5YZC). The compound docks at the neck between F head and stalk, engaging residues in prefusion HR-B but not the fusion peptide. (**D**) Residue-view of the AS-48 (yellow sticks) target site.

As of February 2020, eight small-molecule RSV entry inhibitors have advanced to some stage of clinical testing (Table 1). Common characteristics of most of these drug candidates include exceptionally high potency in the nanomolar or sub-nanomolar range, oral bioavailability, high target specificity, and a very low genetic barrier against viral resistance. Although the chemotypes are structurally distinct, all of the advanced RSV entry inhibitors for which pharmacophores have been solved bind to overlapping druggable sites in the central F cavity near the base of the F head domain (Figure 2), stabilizing the prefusion conformation of the trimer by locking the fusion peptide in place [67]. Accordingly, escape mutations affect residues in the fusion peptides (i.e., F_L141W_) or lining the binding site (i.e., F_D486N_ and F_F488Y_). Resistance hot-spots are located at the interface between prefusion F head and stalk domains (i.e., F_D401E_ and F_D489E_) [68], which contributes to stabilizing the metastable prefusion conformation of F proteins [21,36]. Consistent with the distinct positions of resistance sites, viral escape is thought to reflect distinct principles. Substitutions directly lining the target site or allosterically affecting its shape reduce binding affinity of the ligand without altering the conformational stability of prefusion F [67]. Secondly, mutations in the F head-stalk interface such as F_D401E_ and F_D489E_ can destabilize the prefusion fold, narrowing the window of opportunity for productive interaction of the inhibitor with prefusion F by accelerating the rate of F refolding [68]. This kinetics-based secondary escape mechanism is characterized phenotypically by F hyperfusion activity. 

Two factors in particular exacerbated the anticipated negative impact of rapidly emerging resistance on the clinical potential of RSV entry inhibitors. Firstly, several hot spots have been identified that mediate universal escape from inhibition by all advanced RSV entry inhibitors tested, despite the structural diversity of the different chemotypes [68]. The existence of these pan-resistance sites suggests that it may be challenging to proactively counteract viral escape through synthetic scaffold optimization [69]. Supporting this notion, a large-scale high-throughput drug screen using a recombinant RSV strain carrying a signature pan-resistance mutation did not return any hits blocking F protein activity, although entry inhibitors typically emerge readily and are often pharmacodominant in anti-RSV drug screens [70,71]. Secondly, studies in the mouse RSV pathogenesis model have demonstrated early that signature pan-resistance mutations such as F_D401E_ do not mandatorily coincide with viral attenuation in vivo [68], raising substantial concern that entry inhibitor-resistant RSV may emerge rapidly in the field, and remain pathogenic and possibly able to spread. 

Beyond RSV, very few pneumo- or paramyxovirus entry inhibitor candidates have been subjected to advanced structural characterization of the target docking pose and none has progressed to clinical testing. An exception is a chemically well-behaved small-molecule MeV inhibitor, AS-48, which potently blocks MeV F protein-mediated membrane fusion [61,72]. Primary resistance hot spot to AS-48 is F residue N462 [73], which locates to the non-covalent interaction network that affixes the base of the prefusion F head domain to the HR-B derived stalk (Figure 2). Resolution of this intramolecular interface was demonstrated to be a prerequisite for F refolding [74] and drug-resistant MeV F was hyperfusogenic [73,75], illuminating a conserved role of this microdomain in controlling the conformational stability of prefusion F proteins. Ultimate confirmation that AS-48 blocks MeV entry through locking F in a prefusion conformation came from an MeV F_M94G/N462S_ double mutant, which carries an additional substitution in a defined cavity formed by the F_2_ subunit and depends on dose-dependent stabilization by AS-48 to maintain an intracellular transport-competent conformation [73]. Once displayed at the cell surface, the F_M94G/N462S_/AS-48 complex can be efficiently stimulated by MeV H upon receptor binding, underscoring that the compound indeed preserves a fusion-competent fold of F. 

The mechanistic characterization of AS-48 mode of action was confirmed through a high-resolution crystal structure of prefusion MeV F complexed with the inhibitor, which posited the compound at the base of the central F cavity in direct proximity of residue 462 [35]. Despite an overall similarity of the docking poses of RSV and MeV F inhibitors, only the former physically engage residues in the fusion peptides. In contrast, AS-48 docks distal from the fusion peptide and interacts exclusively with residues lining the central F cavity, presumably reflecting family-specific differences in the geometry of the prefusion F head domain between pneumo- and paramyxoviruses (Figure 2). 

### 3.2. Druggable Sites and Neutralizing Epitopes

**Figure 3:**
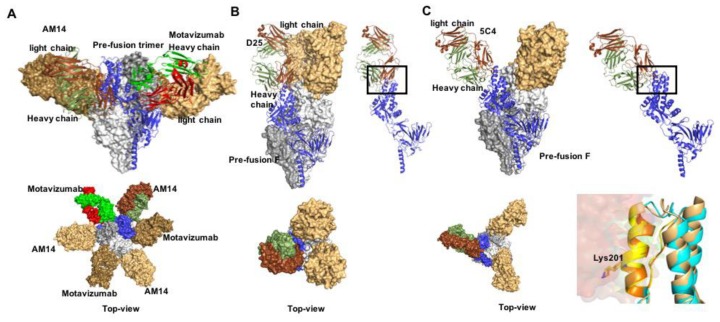
Prefusion RSV F in complex with nAbs. (**A**) Docking poses of motavizumab (red and green) and AM14 (brown and smudge), bound to prefusion F. Both nAbs recognize quaternary epitopes (PDB 4ZYP) and bind the trimer alternatively (lower panel). (**B**,**C**) nAbs D25 and 5C4 (PDB 4JHW and 5W23, respectively) docked to the apex region of the prefusion RSV F protein trimer (antigenic site Ø). (**D**) Antigenic site Ø shown in orange and yellow. Mutation at position 201 mediates resistance.

**Figure 4:**
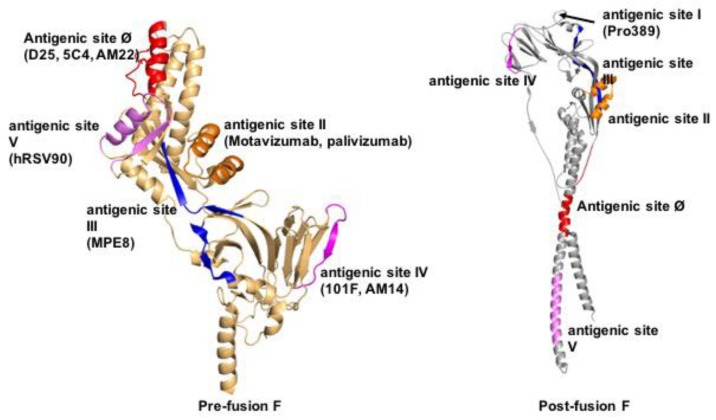
Defined antigenic epitopes in pre- (left) and/or postfusion (right) RSV F. Shown are antigenic sites Ø and I-V. Sites Ø and V are prefusion F-specific, sites II-IV are present in both pre- and postfusion F, and site I is formed in postfusion F only. Ribbon representations of single RSV F monomers are shown for clarity, residues forming the individual epitopes are highlighted.

**Figure 5:**
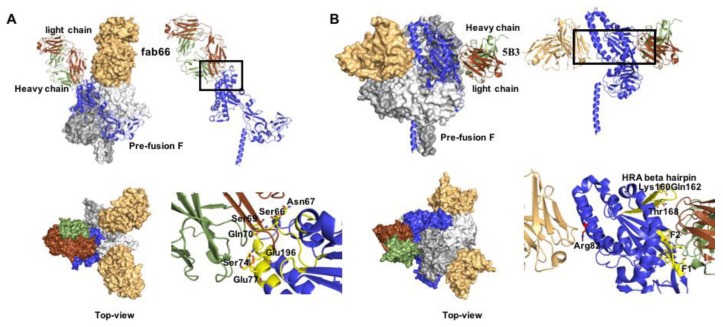
Co-crystal structures of prefusion NiV F with nAbs fab66 and 5B3. (**A**) Side and Table 66. bound to the apex region of the NiV prefusion F trimer (PDB 6T3F). Right panels show a close-up of the epitope, interacting residues are denoted as yellow sticks. (**B**) Side and top view of 5B3 bound to the lateral site of prefusion NiV F (PDB 6TYS). Two F monomers are engaged simultaneously in the interaction. Right panels: both heavy and light chains (depicted in green and brown, respectively) interact with the F protein.

Structures of F proteins complexed with neutralizing antibodies have been solved for a number of pneumo- and paramyxovirus family members including RSV (Figure 3), HPIV3, and NiV. Distinct antigenic sites were identified on RSV F (Figure 4): conformation-dependent site Ø that is located at the apex of prefusion F and a dominant target for nAbs [36,76,77,78]; sites II and IV that are present in both the pre- and postfusion F conformations [67,79]; site III that likewise exists in both F conformations, but undergoes rearrangement of secondary structure elements forming the epitope [80,81]; site V located between sites Ø and III on prefusion F [82,83]; and post-fusion F site I [84,85]. A cryo-EM structure of HPIV3 F complexed with nAb PIA174 likewise locates the binding site to the apex of the prefusion F trimer, establishing contact with residues of all three F protomers [37]. Underscoring that membrane-distal domains of the F proteins are most immune-accessible, two NiV F nAb complexes were recently reported that locate the binding sites at the apex of prefusion F [86] and an adjacent quaternary epitope [87], respectively (Figure 5). The binding sites of these antibodies are conserved in hendra virus F, resulting in good cross-neutralization. 

Naturally, the druggable site near the base of the central prefusion F cavity is sterically not accessible for antibody binding. Pharmacodominance of this site for all highly potent small molecule entry inhibitors characterized so far is nevertheless remarkable, since mapping of nAbs has revealed this diverse set of alternative sites in membrane-distal regions of the F head that, when engaged, stabilize the F prefusion fold. The current lack of small molecule ligands directed against these antigenic sites presumably directly reflects the fundamental differences in the nature of binding sites and mode of target engagement by antibodies and small molecule drugs. Typically, protein-protein interfaces including those between antigen-antibody are flat in geometry, span a large internal surface (the footprint of an antibody is approx. 1000 to 2000 Å^2^), and include numerous polar and hydrophobic interactions [88]. By comparison, small molecules cover at most 300–500 Å^2^ of a protein surface and target sites show concavity, ensuring favorable contacts with multiple sites of the ligand. Although it is therefore challenging to recapitulate the effect of nAbs with small molecules, functional mimicry can be achieved when hot-spot residues on the protein surface are known that contribute overproportionally to the interaction [89]. However, the promise of superior pharmacokinetic properties of small molecule drugs, greater flexibility of administration through different routes, and superior stability and cost-effectiveness has revitalized efforts to replace antibodies with confirmed therapeutic impact with small molecules that mimic antibody binding and functionality [90]. Proof-of-concept for the clinical potential of the approach comes, for instance, from attempts of small molecule-targeting of the PD-1/PD-L1 pathway for cancer therapy [91] and the nAb epitope-informed design of a small molecule alternative to a broadly neutralizing anti-influenza virus antibody directed against the HA stem domain [92]. Given the dramatic expansion in high-resolution structural insight in productive nAb-F protein interaction in recent years, it will be exciting to see whether this knowledge can be equally harnessed for the structure-guided development of new classes of pneumo- and paramyxovirus entry inhibitors. 

### 3.3. Clinical Efficacy of Small Molecule F Protein Inhibitors

Of the small molecule RSV entry inhibitors that have entered the clinical testing phase so far, five have advanced to efficacy testing (Table 1). The majority of these phase II trials employed an established human challenge model of RSV infection that is based on experimental infection of adult healthy volunteers and thus provides a framework to assess treatment under controlled, predefined conditions [93]. Primary efficacy end point of the model was typically shed virus load in nasal swab samples, secondary end points included total mucus weight produced during the acute infection period and symptom scores. Currently available trial outcome data show that three of the entry inhibitor candidates tested, presatovir (GS-5806) [94], RV521 [95], and JNJ-53718678 [96], reduced viral burden, shortened disease duration, and/or alleviated secondary clinical signs. 

Presatovir was subsequently advanced to phase IIb trials in hospitalized patients suffering from RSV infection and in lung and hematopoietic stem cell transplant recipients [97,98,99]. Treatment was overall well-tolerated with minimal adverse effect, but presatovir disappointed overall in these much larger patient groups with confirmed RSV infections, despite its encouraging original performance in the human challenge model. The compound failed to significantly reduce virus load in any of the patient groups tested. Moreover, presatovir had no impact on the duration of hospitalization in the adult patient cohort [97], did not alleviate disease symptoms or improve lung function in the lung transplant recipients [98], and did not lower the rate of respiratory failure or reduce overall mortality in the stem cell transplant recipients [99]. 

Although the antiviral impact of presatovir was marginal in these trials, substitutions in signature resistance microdomains (i.e., at fusion peptide residues 127, 138, 140, and 141, at residues 399–401, and at residues 486–488) emerged in up to 11.2% of patients in treatment groups, but were absent from placebo-treated subjects [100]. Patients in which resistant viruses emerged experienced smaller reductions in viral load, but clinical outcome was similar to that in subjects without viral escape, indicating that resistant viruses replicate efficiently in vivo but do not cause enhanced disease. These clinical data thus confirmed the earlier evidence provided by resistance testing in RSV animal models [68], underscoring that multiple molecular routes to acquire robust resistance to current entry inhibitors are available and that resistant RSV variants remain pathogenic in vivo. 

### 3.4. Perspectives for Pneumo- and Paramyxovirus Entry Inhibitors 

The disappointing outcome of the four presatovir phase IIb clinical trials certainly casts major doubt on the clinical potential of all currently pursued advanced F protein entry inhibitors, especially when considering the overlapping binding sites of the different chemotypes, similar mechanisms of activity, and existence of several pan-resistance hot-spots. Given the mechanistic similarities and the originally encouraging performance of presatovir in the human RSV challenge model, there is little reason to assume that the alternative entry inhibitor scaffolds that have been successfully tested in the challenge model may fare better in phase IIb trials. High-resolution structural model of entry inhibitors with prefusion F have greatly advanced the appreciation of the molecular mechanism of fusion inhibition but have not yet suggested a path to how viral pan-resistance could be counteracted through proactive ligand engineering. Despite sub-nanomolar potency in cell culture assays and initially very attractive drug profile properties, therapeutic targeting of the base of the prefusion F protein cavity may ultimately not be the most effective approach to address the RSV problem. 

Alternatively, allosteric and substrate-analog inhibitors of the RSV polymerase complex have been identified [70,101,102,103,104,105], some of which likewise showed promising drug-like properties and potently blocked virus replication in cell culture and in vivo. Ultimately, the therapeutic targeting of the polymerase may be more fruitful for two main reasons [106]: (i) all enzymatic functions of the viral polymerase complex, initiation of RNA synthesis, RNA elongation, mRNA capping, and cap methylation, must be performed multiple times to complete an individual viral replication cycle, providing—in contrast to entry inhibition—repeated opportunities for interference, and (ii) polymerase blockers suppress not only the synthesis of structural virion components directly, but also heighten virus susceptibility to the innate host antiviral response, since they prevent the expression of viral non-structural immune-modulatory proteins that are essential for viral pathogenesis [107,108]. 

Ultimately, combining entry inhibitors with mechanistically distinct antivirals such as polymerase inhibitors may be the most powerful strategy. However, heightened developmental costs associated with this approach will affect economic viability, and entry inhibitors could lose all impact long-term, should resistant RSV transmit efficiently and viral strains emerge in circulation that carry pre-existing resistance mutations. 

## Figures and Tables

**Table 1:** Small molecule RSV entry inhibitors in clinical phase.

Candidate ID	Sponsor	Phase	Outcome	Trial ID
GS-5806 (Presatovir)	Gilead Sciences	Phase IIb	well tolerated, did not achieve primary endpoints	NCT02254421
GS-5806 (Presatovir)	Gilead Sciences	Phase IIb	well tolerated, did not achieve primary endpoints	NCT02534350
GS-5806 (Presatovir)	Gilead Sciences	Phase IIb	well tolerated, did not achieve primary endpoints	NCT02135614
GS-5806 (Presatovir)	Gilead Sciences	Phase IIa	reduction in RSV load	NCT01756482
RV521	ReViral Ltd.	Phase II	estimated completion October 2021	NCT04225897
MDT-637	MicroDose Therapeutx, Inc	Phase I	well tolerated	NCT01556607
BTA-C585 (Enzaplatovir)	Biota Pharmaceuticals, Inc.	Phase IIa	suspended	NCT02718937
MK-1654	Merck Sharp & Dohme Corp.	Phase IIa	estimated completion April 2020	NCT04086472
AK-0529 (Ziresovir)	Ark Biosciences Inc.	Phase II	completed	NCT02654171
BTA9881	Biota Scientific Management Pty Ltd.	Phase I	discontinued	NCT00504907
JNJ-53718678	Janssen Research & Development, LLC	Phase II	estimated completion November 2020	NCT03656510
